# 4D flow MRI assessment of right atrial flow patterns in the normal heart – influence of caval vein arrangement and implications for the patent foramen ovale

**DOI:** 10.1371/journal.pone.0173046

**Published:** 2017-03-10

**Authors:** Jehill D. Parikh, Jayant Kakarla, Bernard Keavney, John J. O’Sullivan, Gary A. Ford, Andrew M. Blamire, Kieren G. Hollingsworth, Louise Coats

**Affiliations:** 1 Institute of Cellular Medicine, Newcastle University, Newcastle upon Tyne, United Kingdom; 2 Department of Congenital Cardiology, Freeman Hospital, Newcastle upon Tyne, United Kingdom; 3 Institute of Cardiovascular Sciences, University of Manchester, Manchester, United Kingdom; 4 Institute of Genetic Medicine, Newcastle University, Newcastle upon Tyne, United Kingdom; 5 Medical Sciences Division, University of Oxford, Oxford, United Kingdom; Vanderbilt University Medical Center, UNITED STATES

## Abstract

**Aim:**

To investigate atrial flow patterns in the normal adult heart, to explore whether caval vein arrangement and patency of the foramen ovale (PFO) may be associated with flow pattern.

**Materials and Methods:**

Time-resolved, three-dimensional velocity encoded magnetic resonance imaging (4D flow) was employed to assess atrial flow patterns in thirteen healthy subjects (6 male, 40 years, range 25–50) and thirteen subjects (6 male, 40 years, range 21–50) with cryptogenic stroke and patent foramen ovale (CS-PFO). Right atrial flow was defined as vortical, helico-vortical, helical and multiple vortices. Time-averaged and peak systolic and diastolic flows in the caval and pulmonary veins and their anatomical arrangement were compared.

**Results:**

A spectrum of right atrial flow was observed across the four defined categories. The right atrial flow patterns were strongly associated with the relative position of the caval veins. Right atrial flow patterns other than vortical were more common (p = 0.015) and the separation between the superior and inferior vena cava greater (10±5mm versus 3±3mm, p = 0.002) in the CS-PFO group. In the left atrium all subjects except one had counter-clockwise vortical flow. Vortex size varied and was associated with left lower pulmonary vein flow (systolic r = 0.61, p = 0.001, diastolic r = 0.63 p = 0.002). A diastolic vortex was less common and time-averaged left atrial velocity was greater in the CS-PFO group (17±2cm/sec versus 15±1, p = 0.048). One CS-PFO subject demonstrated vortical retrograde flow in the descending aortic arch; all other subjects had laminar descending aortic flow.

**Conclusion:**

Right atrial flow patterns in the normal heart are heterogeneous and are associated with the relative position of the caval veins. Patterns, other than ‘typical’ vortical flow, are more prevalent in the right atrium of those with cryptogenic stroke in the context of PFO. Left atrial flow patterns are more homogenous in normal hearts and show a relationship with flow arising from the left pulmonary veins.

## Introduction

The right atrium is a dynamic, complex structure with multiple functions that evolve during life. In the fetus, it directs nutrient-rich blood to the left heart via the foramen ovale; whilst postnatally it functions as a reservoir, a conduit and a pump to deliver deoxygenated blood via the right ventricle to the pulmonary circulation.

The morphology and function of the right atrium is now well described but there is limited literature describing the nature of blood flow within it. The accepted pattern, reported in case studies, is that of a clockwise vortex with a counter-clockwise vortex observed in the left atrium [1]. Time-resolved, three-dimensional velocity encoded magnetic resonance imaging (4D-flow MRI) has recently emerged as a powerful tool for qualitative and quantitative analysis of blood flow and provides an opportunity to investigate right atrial flow dynamics [2].

The foramen ovale, which is universally present in fetal life, persists postnatally in a quarter of the population [3]. Various clinical syndromes including cryptogenic stroke, decompression sickness, platypnoea orthodeoxia and more controversially migraine have been postulated to arise as a result of paradoxical movement of blood from right to left atrium via a patent foramen ovale (PFO) [4–7]. The size of the PFO and degree of shunt may be influential but these findings are disputed [8,9]. We hypothesize that patency of the foramen ovale and thus susceptibility to paradoxical flow between the atria may relate to variations in flow patterns within the right atrium.

The purpose of this study was to investigate atrial flow patterns in the normal adult heart, primarily to establish whether right atrial flow is always vortical and if not, whether absence of vortical flow may be associated with patency of the foramen ovale in the setting of a related clinical syndrome. In addition we report left atrial and aortic flow, as previous studies have suggested they may be influential in the evolution of cryptogenic stroke in the patient with patent foramen ovale [10–12].

## Materials and methods

### Study population

Thirteen subjects (40.4 years, range 25–50 [male: n = 6, 39.8 years, range 25–48, female: n = 7, 40.8 years, range 35–50]) with normal trans-thoracic echo were prospectively recruited between May 2013 and May 2015. All control subjects were recruited by advertisement within the Newcastle Hospitals NHS Foundation Trust. Also, thirteen subjects (40.0 years, range 21–50 [male: n = 6, 39.7 years, range 21–50, female: n = 7, 40.3 years, range 33–50]) with cryptogenic stroke or transient ischaemic attack and patent foramen ovale (CS-PFO) on trans-oesophageal echocardiography were recruited. Exclusion criteria included age under 18 or over 55 years or contraindication to magnetic resonance imaging (MRI). Newcastle and North Tyneside 2 Ethics committee approved the study (12/NE/0140). All participants gave informed written consent.

CS-PFO subjects had no other identifiable cause for their stroke event and had been referred for consideration of PFO closure and consecutively recruited to this study. Vascular risk factors were assessed at the time of attendance for 4D flow imaging.

### Transoesophageal echo

Trans-oesophageal echocardiography was performed, as per standard clinical protocols in the CS-PFO group, with local anaesthesia under light sedation. The diameter (maximal separation of the septum primum and secundum at rest) and length (maximal overlap of the septum primum and secundum at rest) of the PFO tunnel, presence of an atrial septal aneurysm (excursion >10 mm) and Eustacian valve prominence were recorded [13].

### Magnetic resonance imaging

MRI was performed at 3.0 Tesla (Achieva, Philips Best, The Netherlands) with a 6 channel cardiac array. Multi-slice, multi-phase FFE cine scans in three orthogonal planes were acquired during free breathing for an anatomical overview and to facilitate subsequent planning. Retrospectively-gated steady-state free precession sequences were acquired during a single breath-hold in the vertical and horizontal long axis, short axis and 4-chamber views [14]. 4D flow MRI was performed using a retrospectively ECG-gated and respiratory gated turbo field echo sequence (TR/TE/flip: 6.3ms/3.7ms/8°, VENC: 150m/s, FOV: 240mm(antero-posterior)x240mm (foot-head)x142mm(left-right), spatial resolution: 1x1x1mm^3^, temporal resolution: 50-55ms, 20 phases, SENSE factor 2 and turbo factor 2). A respiratory navigator was employed to reduce motion artifact (navigator efficiency was 60–70%, sequence duration 18.8±3.5 minutes) [15]. Phase errors introduced by eddy currents and Maxwell terms were corrected during the reconstruction process [15]. Eight subjects (four in each group) underwent repeat scans on a separate occasion (9±8 weeks later) for reproducibility.

### Data analysis

**Fig 1:**
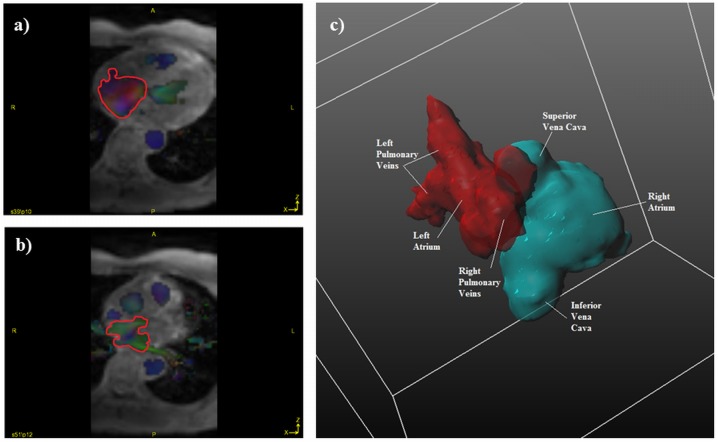
Manual identification in a control subject of a) right atrial and b) left atrial regions of interest (red contours) in the axial plane (depicted during late ventricular systole/atrial filling phase) colour coded according to direction of the flow and c) generation of a three dimensional time-averaged phase contrast angiogram viewed from the right posterior aspect.

**Fig 2:**
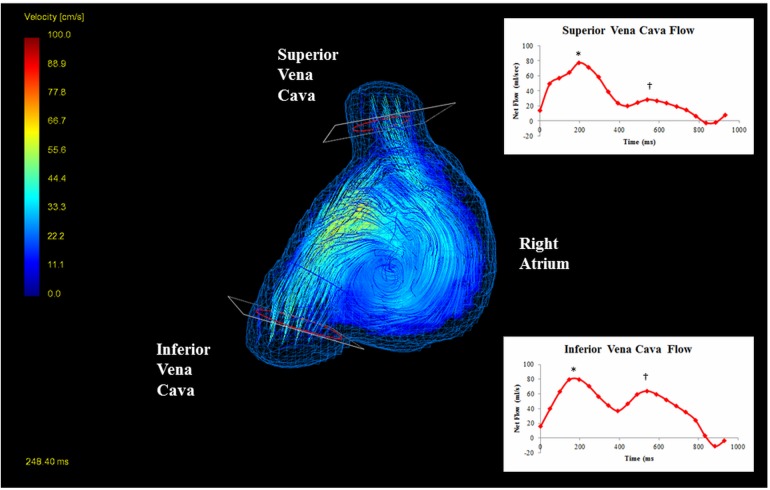
Streamline visualization (velocity encoded) of ‘typical clockwise’ right atrial vortex during late ventricular systole/atrial filling phase in a control subject viewed from the right sagittal aspect, demonstrating positioning of the planes and contours in the caval veins and associated flow waveform across the cardiac cycle (*peak systolic flow, _†_peak diastolic flow, note negative SVC flow reflects directionality; flow magnitude only is reported in the results).

4D flow datasets were analyzed in GTFlow v2.2 (Gyrotools, Zurich, Switzerland) by a single observer blinded to the subject’s clinical details. The atria were segmented using the flow data by manually defining regions of interest in successive axial slices at each time phase **(Fig 1A and 1B)**. A three dimensional time-averaged phase contrast angiogram was generated to produce an approximation of the atrial geometry and facilitate orientation and interpretation of the flow data **(Fig 1C)**. Streamlines were generated within each region to visualize flow patterns **(Fig 2)**. The gross atrial systolic and diastolic flow patterns were described.

#### Right atrium

**Fig 3:**
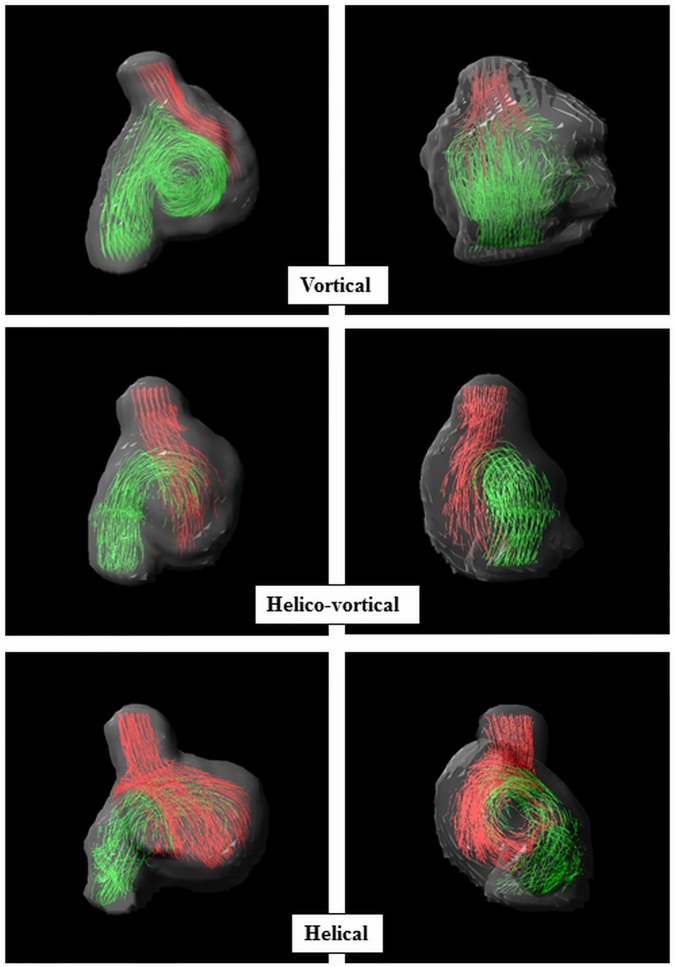
Right atrial systolic flow patterns (streamlines) viewed from the right sagittal (left hand column) and anteroposterior (right hand column) projections during late ventricular systole/atrial filling phase. SVC flow (red) and IVC flow (green).

The manually segmented right atrial region included 4 slices of superior vena cava (SVC) and inferior vena cava (IVC) flow and extended to the tricuspid valve annulus. In the right atrium, vortical flow, as has previously been reported, was defined as a clockwise forward turning vortex with the central core composed of IVC flow and the SVC flow entrained on the outside (Fig 3 top and S1A Video) [2]. Helico-vortical flow was used to describe a vortex composed solely of IVC flow with SVC flow passing laterally and twisting around it in a helical fashion (Fig 3 middle and S1B Video). Helical flow occurred when IVC flow passed medially and SVC flow laterally, with no primary vortex, and curled together in a helical manner (Fig 3 bottom and S1C Video). Number and duration of vortices were recorded. When multiple vortices were present, duration of the dominant vortex was measured. Long and short axis dimensions of the right atrium were measured at end systole in the four-chamber cine view to assess right atrial size. The ratio of these dimensions was calculated as a surrogate measure of sphericity [16].

Planes were positioned, in the angiogram generated from the previous manual segmentation, orthogonal to the caval veins at their junction with the right atrium (the notional point at which a change in vessel calibre was noted as the vein entered the atrium). A contour was placed in each orthogonal plane and adjusted for vessel motion during the cardiac cycle. The flow waveform at each contour was plotted and peak systolic and diastolic flow measured (**Fig 2**). Time-averaged flow was also obtained. Time-averaged velocity within the manually segmented right atrial volumes was also measured.

The relative position of the caval veins was assessed by comparing the co-ordinates of the points at which the centerlines of the respective caval vein flows intersected with the orthogonal planes placed at the junction with the right atrium (S1 Fig).

#### Left atrium

The manually segmented left atrial region included a short length of each pulmonary vein (approximately 1cm) and extended to the mitral valve annulus. Left atrial vortex size was measured at peak systole and diastole using the 4D flow MRI data. A plane was placed orthogonal to the vortex core and the radii of the major and minor axes of the ellipsoidal vortex were measured in order to calculate the cross-sectional area. Duration of the vortex was assessed visually: the vortex was defined as being present when the flow pattern formed a complete circle/ellipse. Left atrial area at end systole in the two-chamber cine view was measured as the best surrogate for left atrial volume [17].

Planes were positioned, using the angiogram generated from the previous manual segmentation, orthogonal to the pulmonary veins at their junction with the left atrium. A contour was placed in each orthogonal plane and adjusted for vessel motion during the cardiac cycle. The flow waveform at each contour was plotted and peak systolic and diastolic flow measured (**Fig 2**). Time-averaged flow was also obtained. Time-averaged velocity within the left atrial volumes was also measured.

#### Aortic arch

Aortic flow was inspected in view of previous reports suggesting it may be implicated in the pathophysiology of cryptogenic stroke [11, 12].

### Reproducibility

Four CS-PFO subjects and four controls underwent repeat scans. Inter-observer and intra-observer analysis was assessed on the eight original scans. For assessment of inter-observer variability, data was independently analyzed by a second observer again blinded to the subject’s clinical details. Intra-observer and scan-scan reproducibility was assessed by a single observer with a one-month gap to eliminate recall bias.

Atrial flow patterns, time-averaged caval flow, time-averaged pulmonary venous flow and time-averaged atrial velocity were each individually compared for each type of reproducibility.

### Statistics

Data were analyzed in Mini-tab (version 16, Minitab Ltd, Coventry, UK). Age is expressed as mean and range. Continuous variables are expressed as mean ± standard deviation. Normality was assessed for all continuous variables using the Anderson-Darling test. Continuous data were compared using the two-tailed, unpaired Student t-test. Categorical data were compared using Fischer’s Exact test. Pearson correlation was used to identify possible predictors of left atrial vortex cross-sectional area. Significance was set at<0.05. The inter-scan, inter-observer and intra-observer bias and 95% limits of agreement were separately calculated for time-averaged caval flow, time-averaged pulmonary venous flow and time-averaged atrial velocity using the Bland-Altman method [18].

## Results

### Study population

Baseline demographics are shown in **Tables 1 and 2**. Body mass index, heart rate and systolic BP were comparable between groups. Diastolic BP was higher in the CS-PFO group (92±16 versus 81±8 mmHg, p = 0.037).

**Table 1:** Baseline demographics of subjects and controls including gender, age, physiological parameters and modifiable cardiovascular risk factors.

	CS-PFO (n = 13)	Controls (n = 13)	P value
Male (n)	6	6	NS
Age (years)	40±8	41±7	NS
Ethnicity—White British (n)	12	11	NS
Height (cm)	172±8	170±10	NS
Weight (kg)	72±14	71±15	NS
BMI (kg/m^2^)	24±3	24±3	NS
Heart Rate (beats per minute)	71±12	67±8	NS
Systolic Blood Pressure (mmHg)	141±24	128±12	NS
Diastolic Blood Pressure (mmHg)	92±16	81±8	0.037
Current or ex-smoker	4	1	NS
Hypercholesterolemia	6	0	0.01
Diabetes Mellites	0	0	NS
Family History of Cardiovascular Disease	5	1	0.16

CS-PFO: Cryptogenic Stroke and Patent Foramen Ovale Subjects, BMI: Body Mass Index

**Table 2:** Type of stroke, clinical management and findings of routine investigations in subjects.

	n
**Nature of Event**	
Total Anterior Circulation Infarction	4
Partial Anterior Circulation Infarction	2
Poster Circulation Infarction	2
Lacunar Infarction	3
Ophthalmic Stroke	1
Transient Ischemic Attack	1
**Areas of Infarction on brain imaging**	
Single	7
Multiple	5
None (ophthalmic stroke)	1
**Management (n = 13)**	
Embolectomy	1
Thrombolysis	4
Secondary Prevention	13
**Holter Monitoring (n = 13)**	
Sinus Rhythm thoughout	13
Occasional superventricular or ventricular extra-systoles	8
Occasional bradycardia or sinus pauses	5
**Thrombophilia Screen (n = 13)**	
Negative	10
Transient borderline positive single result but negative on repeat testing	3
**Vascular Assessment (n = 13)**	
No carotid or vertebral stenosis or dissection	11
Minor irregularities of unknown significance	2

Of the CS-PFO subjects, six were recruited from the Newcastle Hospitals NHS Foundation Trust and seven subjects from approved participant identification centres in the North of England. Eleven CS-PFO subjects had clinical and radiological evidence of cryptogenic stroke. One subject presented with an ophthalmic stroke and had normal brain imaging but evidence of branch retinal artery thrombosis on ophthalmoscopy. One subject’s neurological symptoms resolved within 24 hours, and were thus classified as a transient ischemic attack, but demonstrated multiple minor abnormalities on imaging.

### Transoesophageal echo

The diameter of the PFO tunnel was 3±2mm and tunnel length was 11±5mm. Six subjects had a concomitant atrial septal aneurysm and five patients had a prominent Eustacian valve. One subject had lipomatous hypertrophy of the inter-atrial septum. Four subjects had a severe shunt at rest. No Chiari networks were seen.

### Magnetic resonance imaging

#### Right atrium

**Fig 4:**
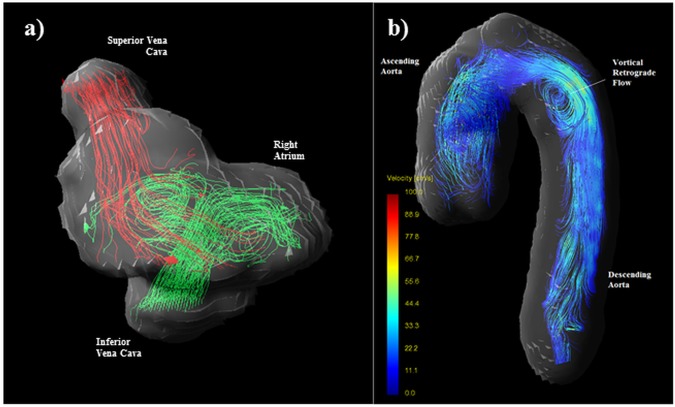
a) Right atrial flow (streamlines) visualized from a right anterior oblique aspect during late ventricular systole/atrial filling phase demonstrating multiple vortices in a CS-PFO subject with a preceding history of lower leg thrombophlebitis and b) retrograde flow (streamlines) in the descending aorta during late systole of CS-PFO patient with a concomitant history of systemic hypertension but no other risk factors.

A spectrum of primary flow patterns were observed and defined as vortical, helico-vortical, helical and multiple vortices as described in the methods (**Fig 3, Table 3**). Five CS-PFO subjects did not have these patterns and exhibited multiple vortices with various patterns observed **(Fig 4A)**. Additional small vortices were identified secondary to the primary flow pattern in the first three groups. No more than two vortices were seen in any scan.

**Table 3:** Right Atrial Size and Flow Characteristics in CS-PFO group and matched controls.

	CS-PFO (n = 13)	Controls (n = 13)	P
**Heart Rate**	67±8	71±12	NS
**RA size and shape**			
RA long axis dimension (mm)	51±9	52±5	NS
RA short axis dimension (mm)	44±3	47±4	NS
RA long:short axis dimension	1.2±0.2	1.1±0.1	NS
**RA Systolic Flow Pattern**			
Vortical	2	8	Vortical versus non-vortical: p = 0.015
Helico-Vortical	5	4	
Helical	1	1	
Multiple Vortices	5	0	
Vortical Duration (ms)	251±69	257±49	NS
Additional Vortex/Vortices	1	4	NS
**RA Diastolic Flow Pattern**			
Vortical	6	10	Vortical versus non-vortical: NS
Helico-Vortical	3	1	
Helical	2	2	
Multiple Vortices	2	0	
Vortical Duration (ms)	248±103	252±100	NS
Time-averaged SVC flow (ml/sec)	40.8±9.7	40.3±14.1	NS
Time-averaged IVC flow (ml/sec)	62.0±17.0	62.5±17.3	NS
**Time-averaged RA Velocity (cm/s)**	17.4±2.7	17.0±1.6	NS
SVC/IVC antero-posterior separation (mm)	16±7	17±6	NS
SVC/IVC right-left separation (mm)	10±5	3±3	0.002

CS-PFO: Cryptogenic Stroke and Patent Foramen Ovale Subjects, RA: Right Atrial, SVC: superior vena cava, IVC: inferior vena cava

Absence of the ‘standard’ vortex during systole and diastole was more common in the CS-PFO group (**Table 3**). Peak systolic and diastolic flow in both SVC and IVC was comparable between CS-PFO subjects and controls. Time to peak systolic and diastolic flow, when adjusted for heart rate also showed no difference between CS-PFO subjects and controls. However, when analysed for flow patterns, non-vortical right atrial flow (helico-vortical, helical and multiple vortices) showed reduced peak systolic flow (135±36 versus 179±45ml/sec, p = 0.02) and a trend to reduced peak diastolic flow (94±29 versus 118±44 ml/sec, p = 0.08) in the IVC. No difference in time-averaged caval flow or time-averaged atrial velocity was found between groups or between flow patterns (**Table 3**).

The position of the caval veins, as assessed by comparing the points at which the centerlines of the respective caval vein flows intersected with the orthogonal planes placed at the junction with the right atrium, was found to be constant in the antero-posterior direction but significantly variable in the right left direction. In the right-left plane the separation between the IVC and the SVC was greater in the CS-PFO group compared to controls (10±5mm versus 3±3mm, p = 0.002). The separation of the caval veins in the right left plane also corresponded to systolic flow patterns (Table 4, S1 Fig).

**Table 4:** Distance between vena cava in the right left plane (judged by comparing points at which centerlines of respective caval vein flows intersected with right atrial junction, S1 Fig) in CS-PFO group and controls.

	n	SVC-IVC separation in RL plane (mm)	p
**Systolic Flow Pattern**			
Vortical	10	1.8±2.5	Vortical versus all other flow types (Helico-vortical, helical and multiplevortices) flow P<0.001
Helico-Vortical	9	7.4±4.5	
Helical	2	11.1±2.4	
Multiple Vortices	5	12.0±5.4	
**Diastolic Flow Pattern**			
Vortical	16	4.6±5.4	Vortical versus all other flow types: P = 0.030
Helico-Vortical	4	7.7±4.5	
Helical	4	10.0±2.4	
Multiple Vortex	2	11.0±9.8	

SVC: superior vena cava, IVC: inferior vena cava

#### Left Atrium

The majority of CS-PFO subjects (11 out of 13) and controls (12 out of 13) had four pulmonary veins. There was no statistical difference between the numbers of pulmonary veins seen between groups. There was also no difference in left atrial size between groups, although it was notable that the CS-PFO group exhibited a greater range of left atrial sizes. All CS-PFO subjects and controls, except one, had ‘typical’ vortical flow within the left atrium, described in literature as counter-clockwise [2]. One control subject had three small systolic vortices, two clockwise and one counter-clockwise. These were located at the orifices of the left upper pulmonary vein, right upper pulmonary vein and right lower pulmonary vein respectively. The presence of a diastolic vortex was less prevalent within the CS-PFO group but this difference did not reach statistical significance (**Table 5**).

**Table 5:** Left atrial, anatomy, size and flow characteristics in CS-PFO group and matched controls.

	CS-PFO (n = 13)	Controls (n = 13)	p
**Heart Rate**	67±8	71±12	NS
**Number of Pulmonary Veins**			
4	11	12	NS
5	2	0	
6	0	1	
**Left Atrial Area—2 Ch (cm**^**2**^**)**	21.4±5.3	20.3±2.5	NS
Time-averaged LLPV flow (ml/sec)	19.3±8.1	15.2±8.4	NS
Time-averaged LUPV flow (ml/sec)	21.8±6.1	20.3±6.1	NS
Time-averaged RUPV flow (ml/sec)	28.3±10.1	27.5±9.9	NS
Time-averaged RLPV flow (ml/sec)	26.8±11.0	22.5±9.9	NS
**Systolic Vortex**	13	12	NS
Duration (ms)	204±47	178±49	NS
Size (mm^2^)	707±519	600±581	NS
Additional systolic vortices	4	6	NS
**Diastolic Vortex**	9	12	NS
Duration (ms)	192±76	186±79	NS
Size (mm^2^)	682±372	573±425	NS
**Time-averaged Left Atrial Velocity (cm/s)**	17±2	15±1	P = 0.048

CS-PFO: Cryptogenic Stroke and Patent Foramen Ovale Subjects, LLPV: left lower pulmonary vein, LUPV:left upper pulmonary vein, RUPV: right upper pulmonary vein, RLPV: right lower pulmonary vein

Whilst there was no difference in time-averaged pulmonary vein flow between groups (**Table 5**), a lower peak diastolic flow was noted within the right upper pulmonary veins of the CS-PFO group (38.1±12.5 versus 54.5±20.1ml/s, p = 0.02). There were no other differences seen in peak systolic or diastolic pulmonary vein flows. Time-averaged left atrial velocity was greater in the CS-PFO group (**Table 5**).

Systolic and diastolic vortex size was variable within both groups (**Table 5**) and correlated with the time-averaged left lower pulmonary vein flow (systolic vortex r = 0.61, p = 0.001, diastolic vortex r = 0.63 p = 0.002 respectively). Systolic and diastolic vortex size also correlated with time-averaged right upper pulmonary vein flow (systolic vortex: r = 0.36, p = 0.072, diastolic vortex r = 0.45, p = 0.043). No other time-averaged pulmonary vein flow was associated with vortical size.

#### Aortic arch

One CS-PFO subject, with a lacunar infarct, hypertension, hypercholesterolaemia and a family history of cardiovascular disease, demonstrated vortical retrograde flow in the descending aortic arch **(Fig 4B)**. In the presence of aortic atheroma this finding has been previously reported as a possible mechanism of cryptogenic stroke when traditional investigations have proven negative [11, 12]. All other subjects demonstrated laminar descending aortic flow.

### Reproducibility

A summary of the reproducibility data derived from the Bland Altman analysis is shown in **Table 6 and S2 Fig**. Observed systolic and diastolic flow patterns in the right and left atrium were entirely reproducible. Variability between and within observers was seen in the measurement of venous flow, which is likely to reflect differences in contour positioning. The wider limits of agreement between scans for these measurements may also reflect the sensitivity of venous flow to differences in physiological conditions. However, there was low bias and time-averaged atrial velocity, which does not depend on contour positioning, showed demonstrated excellent reproducibility.

**Table 6:** Summary Data for Bland-Altman Plots to Characterize Inter and Intra Observer and Scan-Scan Reproducibility of Atrial 4D Flow Measurements.

Reproducibility	Scan-scan		Inter-observer		Intra-observer	
	Bias	95% Limits of Agreement	Bias	95% Limits of Agreement	Bias	95% Limits of Agreement
Time-averaged caval flow (ml/sec)	-1.90	±16.65	0.60	±12.76	-0.31	±7.64
Time-averaged pulmonary vein flow (ml/sec)	-1.01	±14.44	2.08	±9.82	-0.81	±8.14
Time-averaged atrial velocity (cm/s)	-0.45	±2.84	**-**	**-**	**-**	**-**

## Discussion

The present study demonstrates a spectrum of right atrial flow patterns in the structurally normal heart. The flow pattern appears to be associated with the relative arrangement of the caval veins. Atypical (non-vortical) patterns of right atrial flow were more prevalent in those with cryptogenic stroke in the context of PFO compared to controls.

Literature on atrial flow is limited, however the expected pattern that is described in case studies is that of a clockwise vortex in the right atrium and a counter-clockwise vortex in the left atrium when viewed from the front [1]. The presence of separate systolic and diastolic components of the main vortex and additional smaller vortices has also been noted in the left atrium [19, 20]. The effect of morphology on flow has been acknowledged by those seeking to optimize surgical repairs within the heart. However, little work has been done to examine this and its potential consequences in the structurally normal heart [21].

Our findings suggest several distinct patterns of right atrial flow, including vortical, helico-vortical, helical and multiple vortical flow, exist in the structurally normal heart. We have also noted an association between the spatial relationship of the caval veins and the pattern of right atrial flow observed: increased right-left separation of the caval veins, seen more frequently in the CS-PFO group, is associated with loss of the standard vortex and more septally directed IVC flow. The absence of ‘typical’ rotational flow in the right atrium and the deflection of IVC flow on arrival in the right atrium have previously been described in neonates [22]. Rather than being a consequence of imbalanced passive and active right ventricular filling as the authors of [22] suggest, it is likely to be due to the morphological arrangement of the caval veins and perhaps one of the factors that leads to the maintenance of PFO patency in a proportion of the population. This is supported by a case we reported, of a subject with platydeoxia orthopnoea, where the orientation of IVC flow appeared to be associated, uniquely, with reversal of the right atrial vortex and substantial shunting across the PFO [23]. We did not find a gross difference in right atrial size or shape, but the morphology of this chamber is complex with no standard approach to volumetric analysis [16]. Further work will be required to detail the contribution of variation in the chamber morphology to vortex evolution.

Within the left atria, flow patterns were observed to be more homogeneous with variability limited to vortex size influenced by the magnitude of left lower pulmonary vein flow, a finding that is consistent with other studies [24]. Overall time-averaged left atrial velocity was higher in the CS-PFO group, discounting echo findings that left atrial dysfunction similar to that seen in atrial fibrillation may co-exist with PFO [25]. In atrial fibrillation, 4D flow MRI has demonstrated a reduction in time-averaged left atrial velocities [26, 27]. However, lower peak diastolic flow in the right upper pulmonary vein and a tendency to absence of a diastolic vortex was noted in the CS-PFO group. These subtle changes may be of relevance as flow across the PFO typically occurs during diastole. One control subject was noted to have left atrial flow composed of a series of vortices rather than a single dominant one. This subject had flattening of the left atrium with the pulmonary veins entering very laterally aspects such that flow appeared to directly hit that arriving from the opposing pulmonary veins. The aortic root of this subject was mildly dilated; the heart was otherwise entirely normally in terms of chamber dimensions and function.

Recently, MRI studies have implicated retrograde flow from complex atherosclerotic plaques of the proximal descending aorta in the pathophysiology of cryptogenic stroke [11, 12]. Vascular risk factors were present in some of our CS-PFO population, and gross disturbance of descending aorta flow was observed in one. Consideration of this potential mechanism alongside intra-cardiac flow patterns may better elucidate the group in whom the PFO may be pathogenic.

Both groups were well matched, though diastolic blood pressure was higher in the CS-PFO group (92.5±16.2mmHg vs. 81.33±7.4mmHg, p = 0.046). It is possible that this may have influenced time-averaged atrial velocities, however left atrial diastolic velocities were lower in the CS-PFO group. Quantitative measurements reported in this study are consistent with other studies [20, 24]. Since comparison is between groups as opposed to ascribing significance to an absolute value, the relevance of variability arising due to subject and methodological differences is minimized. In this study, we present a qualitative approach for evaluation of complex right atrial vortices in an attempt to identify potential clinical utility. On-going methodological development to obtain a validated method for right atrial vortex quantification and visualization in an automated work flow process will permit wider clinical application. [25, 28–30]. The groups assessed were small and it was not possible therefore to investigate whether features such as size of shunt across the PFO or presence of atrial septal aneurysm were associated with flow pattern. Further work is required to validate these findings in a larger cohort of subjects and to compare with subjects who have incidental PFOs and no history of cryptogenic stroke and others who have syndromes including migraine and decompression sickness.

In conclusion, the spectrum of right atrial flow and its association with the spatial relationship of the caval veins and patency of the PFO, described here, represent an initial step to understanding the complex interaction between morphology and flow patterns in the structurally normal heart potentially with clinical consequences.

## Supporting information

S1 Fig Schematic representation of the right left separation of the superior and inferior vena cava with different right atrial flow patterns. (TIF)

S2 Fig Bland Altman Graphs for a) pulmonary venous flow b) caval vein flow and c) time-averaged atrial velocity
(PDF)

S1 Video **Movies of a) vortical b) helico-vortical and c) helical right atrial flow** [red: superior vena cava flow, green: inferior vena cava flow].
(MP4)
