# Supplementary figures and images for: 4D flow MRI assessment of right atrial flow patterns in the normal heart – influence of caval vein arrangement and implications for the patent foramen ovale
Source: PLoS One. 2017 Mar 10;12(3):e0173046. doi: 10.1371/journal.pone.0173046 (PMC5345792; doi:10.1371/journal.pone.0173046)

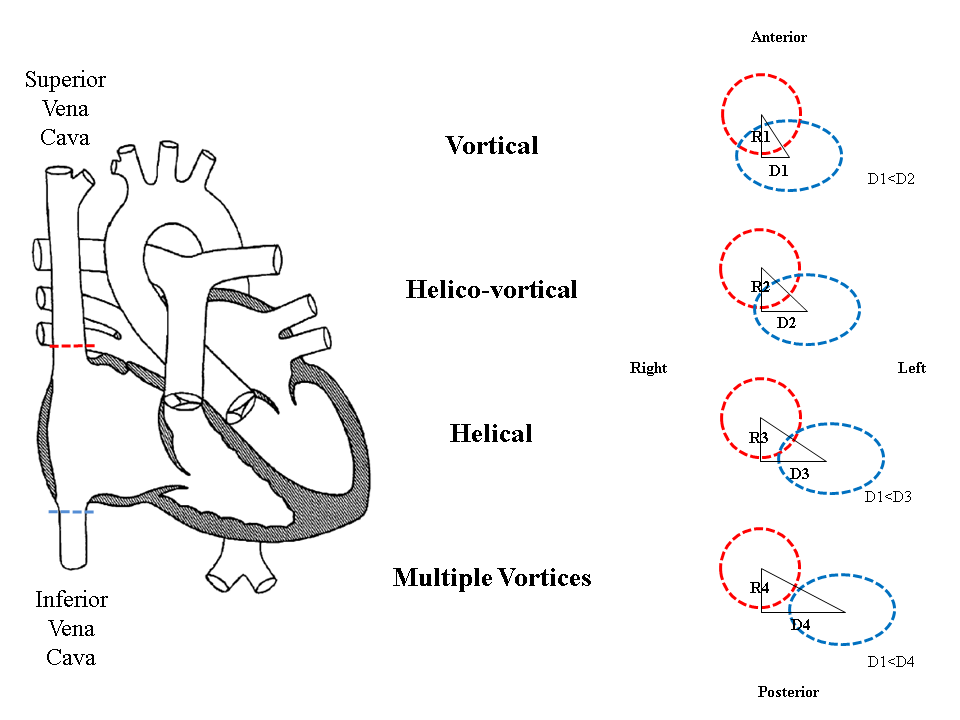

Supplement: S1 Fig — (TIF) [file pone.0173046.s001.TIF]
