# Supplementary material for: 4D flow MRI assessment of right atrial flow patterns in the normal heart – influence of caval vein arrangement and implications for the patent foramen ovale
Source: PLoS One. 2017 Mar 10;12(3):e0173046. doi: 10.1371/journal.pone.0173046 (PMC5345792; doi:10.1371/journal.pone.0173046)

Pulmonary Venous Flow (Intra-observer Variability), ml/sec

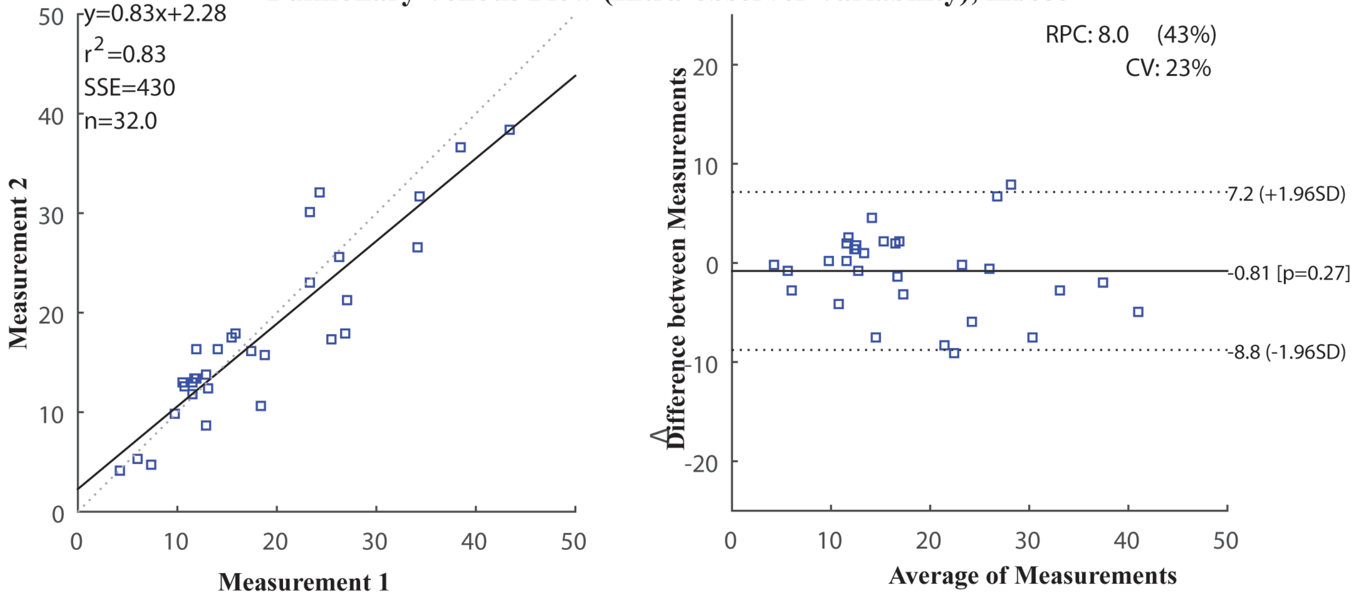

Pulmonary Venous Flow (Inter-observer Variability), ml/sec

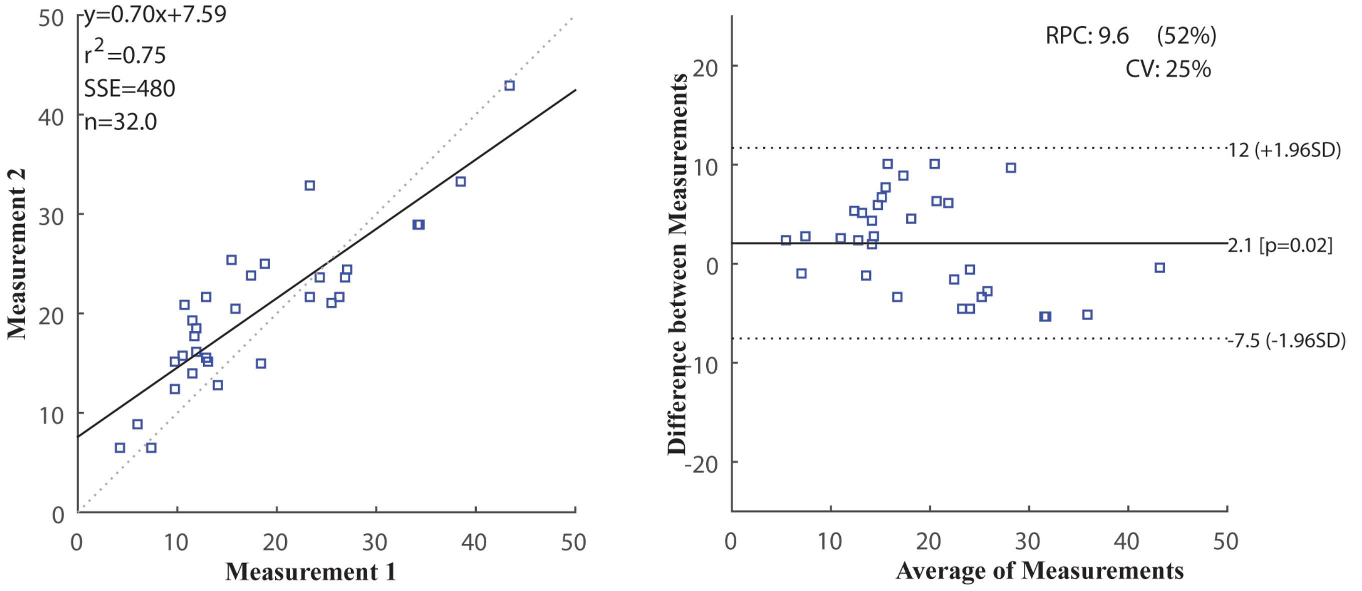

Pulmonary Venous Flow (Scan-scan Reproducibility). ml/sec

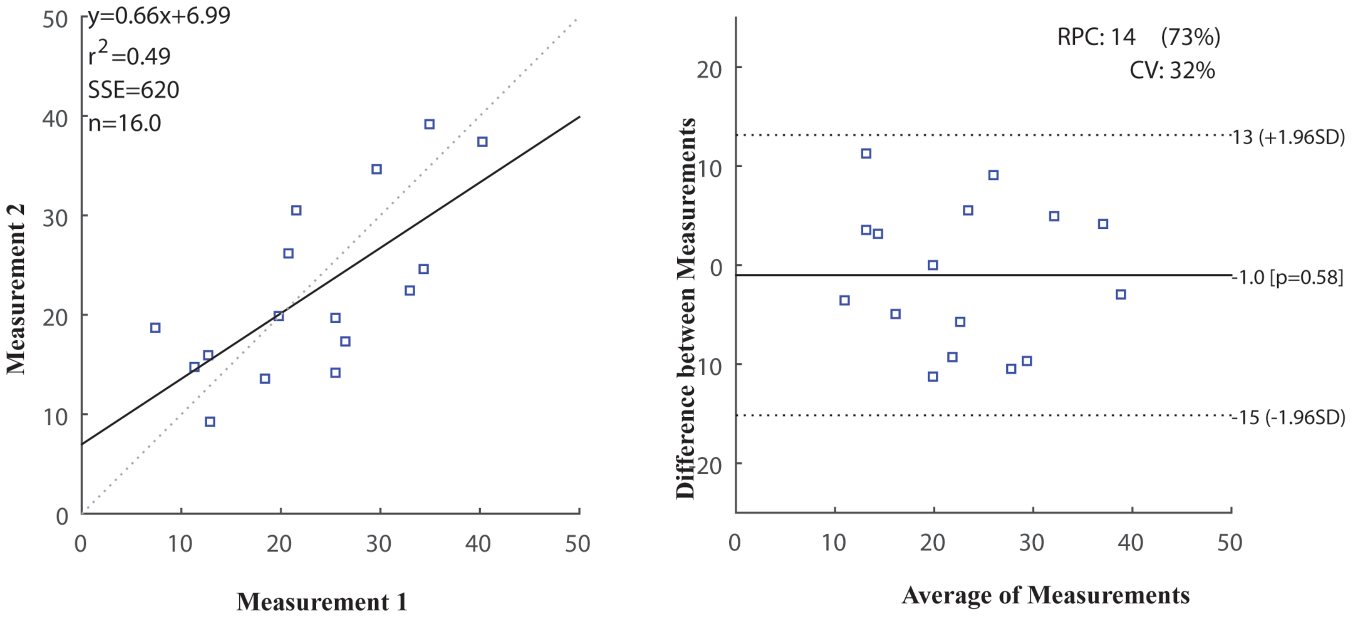

Caval Vein Flow (Intra-observer Variability), ml/sec

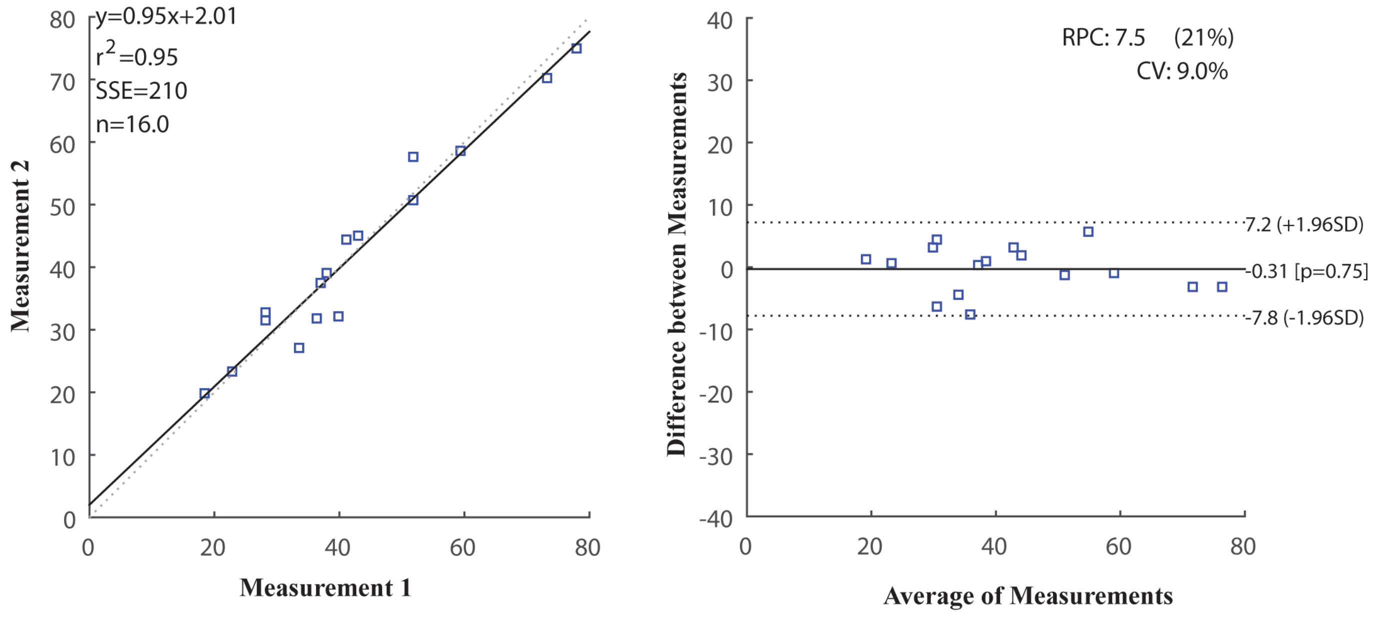

Caval Vein Flow (Inter-observer Variability), ml/sec

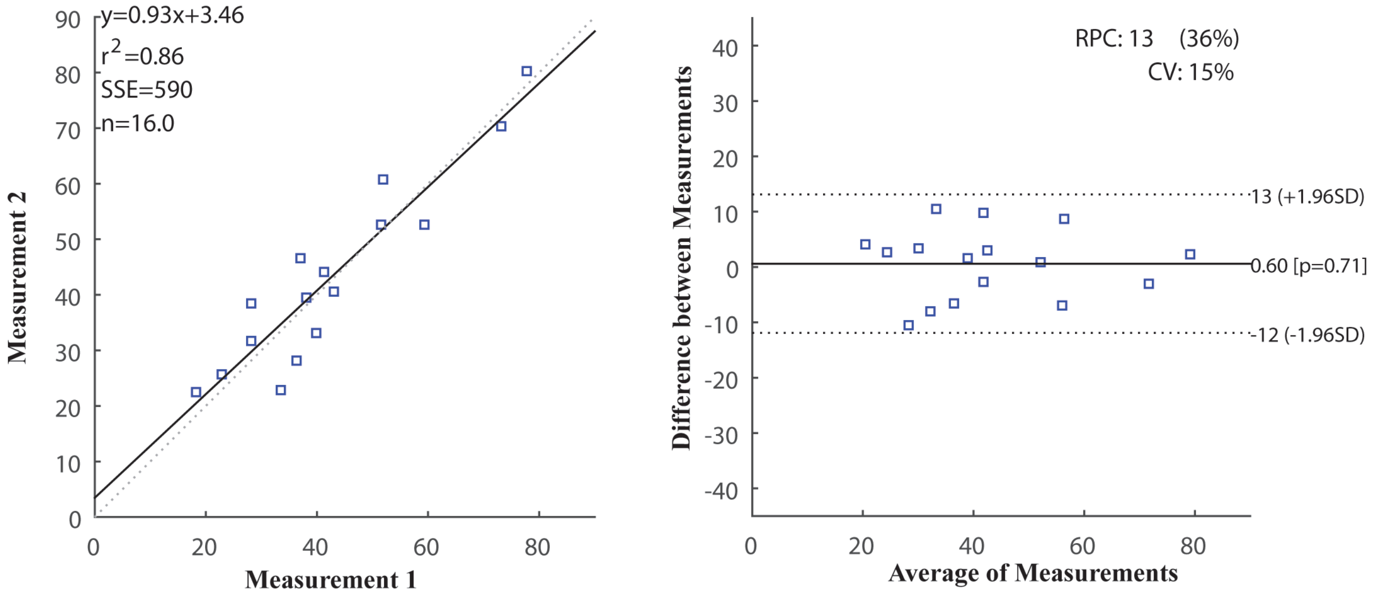

Caval Vein Flow (Scan-scan reproducibility), ml/sec

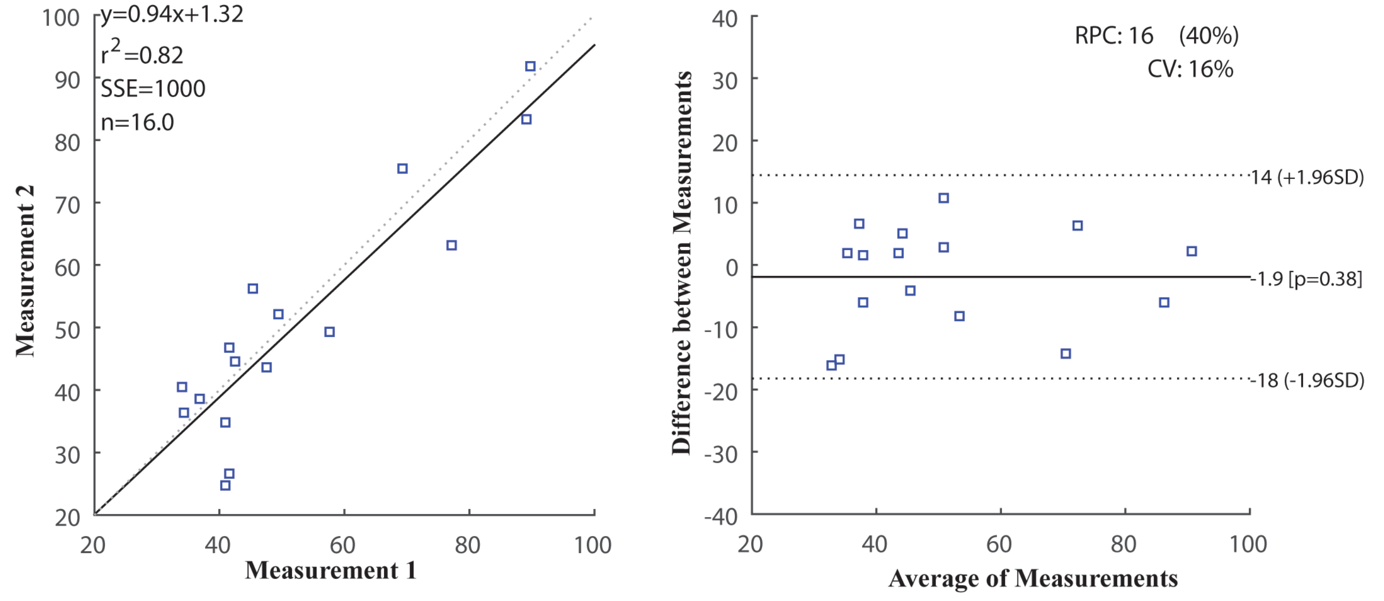

Time-averaged Atrial Velocity (Scan-scan reproducibility), ml/sec

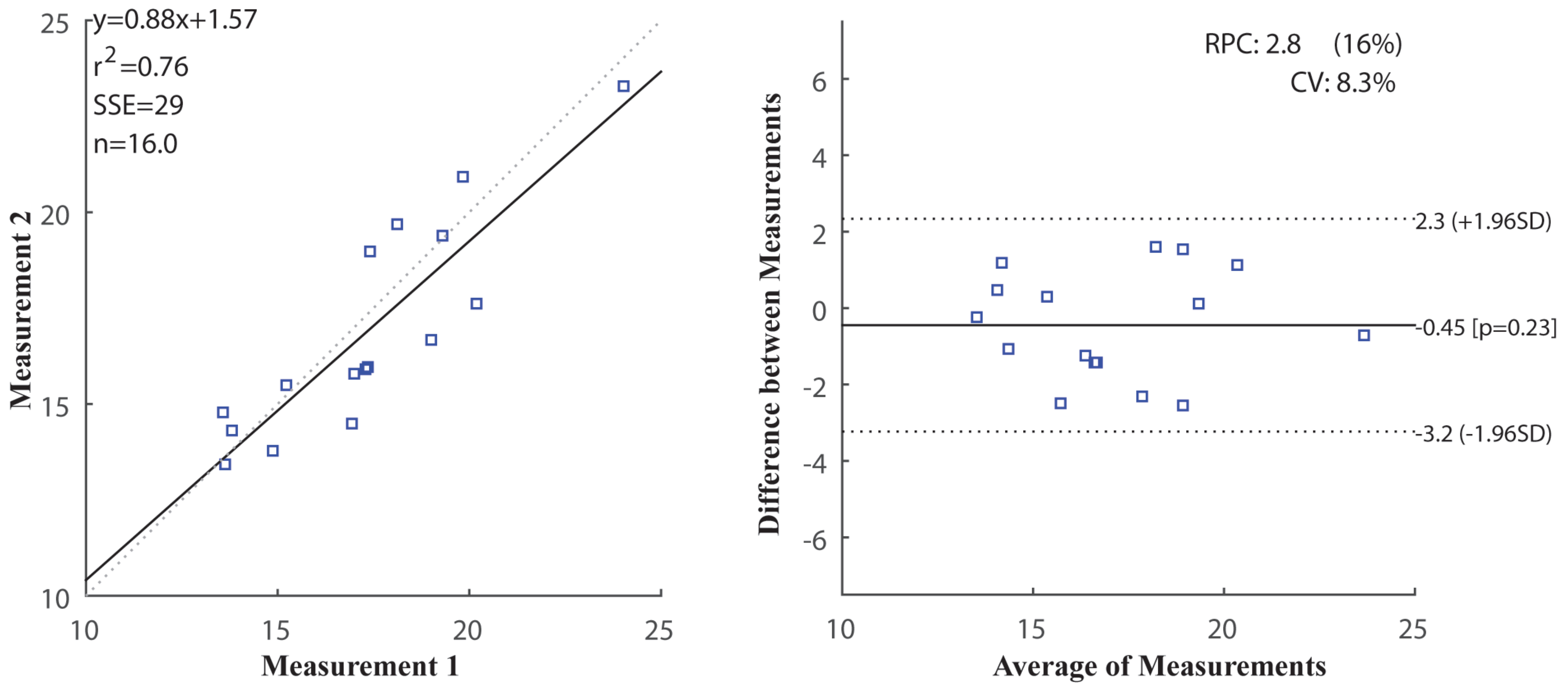

Supplement: S2 Fig — Bland Altman Graphs for a) pulmonary venous flow b) caval vein flow and c) time-averaged atrial velocity (PDF) [file pone.0173046.s002.pdf]
